# New Vertical Handover Method to Optimize Utilization of Wireless Local Area Network in High-Speed Environment

**DOI:** 10.1371/journal.pone.0165888

**Published:** 2016-11-04

**Authors:** Hoe Tung Yew, Eko Supriyanto, Muhammad Haikal Satria, Yuan Wen Hau

**Affiliations:** 1 Faculty of Engineering, Universiti Malaysia Sabah, Kota kinabalu, Malaysia; 2 IJN-UTM Cardiovascular Engineering Centre, Faculty of Biosciences and Medical Engineering, Universiti Teknologi Malaysia, Skudai, Malaysia; 3 Faculty of Biosciences and Medical Engineering, Universiti Teknologi Malaysia, Skudai, Malaysia; 4 IJN-UTM Cardiovascular Engineering Centre, Faculty of Biosciences and Medical Engineering, Universiti Teknologi Malaysia, Skudai, Malaysia; University of Texas at San Antonio, UNITED STATES

## Abstract

In heterogeneous wireless networks, wireless local area network (WLAN) is highly preferred by mobile terminals (MTs) owing to its high transmission bandwidth and low access cost. However, in high-speed environment, handover from a cellular network to a WLAN cell will lead to a high number of handover failures and unnecessary handovers due to the WLAN coverage limitation and will become worse at high speed. A new vertical handover method is proposed to minimize the probability of handover failure and unnecessary handover while maximizing the usage of WLAN in high-speed environment. The simulation results show that the proposed method kept the probability of handover failure and unnecessary handover below 0.5% and 1%, respectively. Compared with previous studies, the proposed method reduced the number of handover failures and unnecessary handovers up to 80.0% and 97.7%, respectively, while the MT is highly mobile. Using the proposed prediction method, the MT can benefit high bandwidth and low network access cost from the WLAN with minimum interruption regardless of speed.

## Introduction

Next-generation wireless networks will be driven by an all-IP-based network infrastructure that provides seamless mobility and ubiquitous access to Internet through heterogeneous wireless networks [1]. In heterogeneous wireless network, wireless local area network (WLAN) is highly preferred owing to its high capacity and low access cost [2, 3]. However, because of limited WLAN coverage, handover from a cellular network to the WLAN may lead to a high number of handover failures and unnecessary handovers when a mobile terminal (MT) is in a high-speed environment because the MT requires less time to cross the WLAN coverage when the speed increases [3, 4].

Handover failure occurs when the MT traveling time within a WLAN (*T*_*WLAN*_) is less than the handover latency from a cellular network to the WLAN (*T*_*i*_). The MT leaves the WLAN coverage before the handover process is executed causing network connection breakdown and service interruption [3–5]. On the other hand, if *T*_*WLAN*_ is equal to or less than the total time of handover to (*T*_*i*_) and out of (*T*_*o*_) the WLAN coverage (*T*_*WLAN*_ ≤ (*T*_*i*_ *+ T*_*o*_)), unnecessary handover occurs [6]. In this situation, the MT does not transmit or receive any data packet via the WLAN because the MT triggers handover out of the WLAN only after completion of handover into the WLAN. Unnecessary handover is undesirable because it wastes network resources [7].

At present, WLAN application is restricted to static or pedestrian navigation environment [8]. For example, in the patient monitoring system using heterogeneous wireless networks proposed by Niyato et al. [2], WLAN was used only for communication within a building such as mall, clinic, hospital, and home. Furthermore, most of the existing handover decision-making algorithms predefined a speed threshold for the WLAN where the MT handovers to the WLAN if and only if the speed of MT is lower than the threshold to prevent unnecessary handover to the WLAN at high mobility. The network-selection algorithms presented in [9–12] defined the WLAN speed threshold at 5 m/s and below. In addition, the fuzzy multi-criteria-based vertical handover algorithm presented by Kaleem et al. [13] and Yew et al. [14] set the rule: “if MT velocity (*v*) is low, then the probability of WLAN rejection is low; else the probability of WLAN rejection is high.”

**Fig 1:**
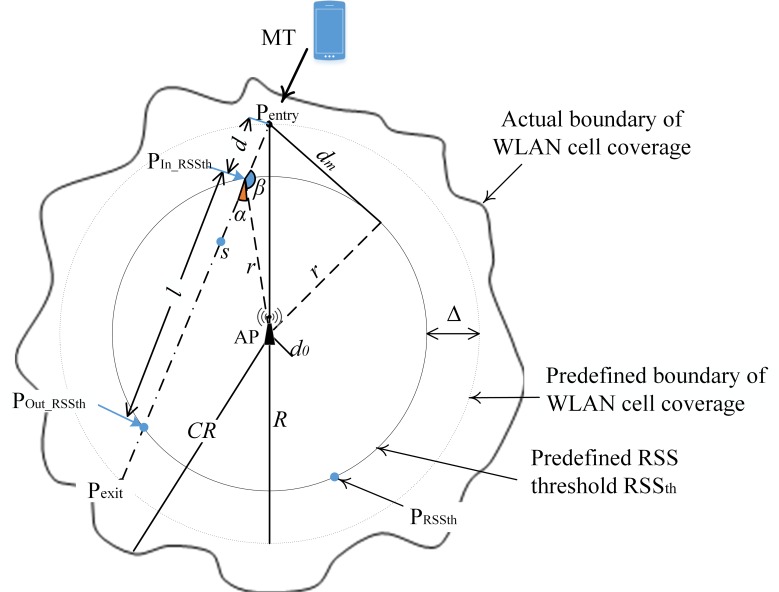
MT traveling trajectory in WLAN cell coverage.

Yan et al. [6] and Hussain et al. [15] presented a traveling distance prediction-based vertical handover scheme to minimize the number of handover failures and unnecessary handovers to WLAN with assumption that MT travels in constant speed within the WLAN coverage. In these schemes, MTs have to take two received signal strength (RSS) sample points within a WLAN cell. The first RSS sample point is at the point where the MT detects a predefined RSS threshold (P_In_RSSth_), and the second sample point (*s*, as shown in Fig 1) can be any point within the WLAN cell (after the MT enters the WLAN cell). The trigonometric function is then applied to predict the MT traveling distance within the WLAN cell. The MT triggers handover to the WLAN if and only if the estimated traveling distance within the WLAN is greater than the distance threshold (*L*_*th*_). The *L*_*th*_ of handover failure presented by Yan et al. [6] (*L*_*thfY*_) and Hussain et al. [15] (*L*_*thfH*_) is expressed as

$$L_{thfY}=2rsin({sin}^{-1}\left(\frac{v(T_{i})}{2r}\right)-\frac{\pi P_{f}}{2})$$ (1)

$$L_{thfH}=\frac{2rtan({tan}^{-1}(\frac{v(T_{i})}{\sqrt{{4r}^{2}-v^{2}{T_{i}}^{2}}})-\frac{\pi P_{f}}{2})}{\sqrt{1+{\left(tan({tan}^{-1}(\frac{v(T_{i})}{\sqrt{{4r}^{2}-v^{2}{T_{i}}^{2}}})-\frac{\pi P_{f}}{2})\right)}^{2}}}.$$ (2)

The unnecessary handovers *L*_*th*_ presented by Yan et al. [6] (*L*_*thuY*_) and Hussain et al. [15] (*L*_*thuH*_) are expressed as

$$L_{thuY}=2rsin({sin}^{-1}\left(\frac{v(T_{i}+T_{o})}{2r}\right)-\frac{\pi P_{u}}{2})$$ (3)

$$L_{thfH}=\frac{2rtan({tan}^{-1}(\frac{v(T_{i}+T_{o})}{\sqrt{{4r}^{2}-v^{2}{{(T}_{i}+T_{o})}^{2}}})-\frac{\pi P_{f}}{2})}{\sqrt{1+{\left(tan({tan}^{-1}(\frac{v(T_{i}+T_{o})}{\sqrt{{4r}^{2}-v^{2}({T_{i}+T_{o})}^{2}}})-\frac{\pi P_{f}}{2})\right)}^{2}}}$$ (4)

where *r* is the WLAN radius, *v* is the measured MT’s velocity, and *P*_*u*_ and *P*_*f*_ denote the user-acceptable probability of unnecessary handover and handover failure, respectively. The results show that both the probability of handover failure and unnecessary handover are kept within the user acceptable value [6, 15]. However, the optimum time to take the second RSS sample point was not considered by the authors. We have found that the larger the time gap between the two RSS sample points is, the lower becomes the accuracy of the prediction result. The time needed by the MT to cover the two RSS sample points is expressed as $t_{s}-t_{P_{In\_RSSth}}$, where *t*_*s*_ is the time of the MT at the second RSS sample point *s* and $t_{P_{In\_RSSth}}$ is the time of the MT at the first sample point P_In_RSSth_ (Fig 1). Therefore, using the methods presented by Yan et al. [6] and Hussain et al. [15], the actual beneficial time (*AT*_*WLAN*_) for the MT should be

$${{AT}_{WLAN}=T}_{WLAN}-(t_{s}-t_{P_{In\_RSSth}})$$ (5)

In this paper, a novel vertical handover method is proposed to improve the accuracy of the traveling-time estimation and to minimize both handover failure and unnecessary handover to the WLAN when the MT is in a high-speed environment.

## Proposed Vertical Handover Method

Fig 1 shows the MT travel trajectory in WLAN cell coverage. We assume that the MT crosses the WLAN cell from an entry point (P_entry_) to an exit point (P_exit_) (as shown in Fig 1) at irregular speed (accelerate or decelerate). The beneficial time (*T*_*WLAN*_) for the MT within the WLAN cell is defined as the time the MT travels from points P_In_RSSth_ to P_Out_RSSth_. The MT would only be able to benefit from the connected WLAN cell if *T*_*WLAN*_ > (*T*_*i*_ *+ T*_*o*_).

In Fig 1, *R* denotes the distance between P_exit_ or P_entry_ and the WLAN access point (AP), P_RSSth_ is any point that falls on the predefined RSS_th_ line, *r* is the distance between P_RSSth_ and AP, and *d* is the MT traveling distance from P_entry_ to P_In_RSSth_. *d* is expressed as

$$d=t_{d}*\frac{v_{e}+v_{R}}{2},\;d\in [\Delta ,d_{m}]$$ (6)

where *t*_*d*_ is time taken by the MT to travel from P_entry_ to P_In_RSSth_, *v*_*e*_ *and v*_*R*_ denotes velocity at P_entry_ and P_In_RSSth_ respectively, which can be measured using an accelerometer or velocity estimation algorithm in [16], *Δ* is the minimum value of *d*, namely, (*R-r*), and *d*_*m*_ represents the maximum *d* value, *$\left(\sqrt{R^{2}-r^{2}}\right)$*. The *R* and *r* values can be determined based on the RSS value measured at points P_entry_ (${RSS}_{P_{entry}}$) and P_In_RSSth_ (${RSS}_{P_{In\_RSSth}}$), respectively, using the log-distance path loss model [17], as expressed by (7).

$$\begin{array}{c} RSS_{P_{entry}}=P_{TX}-PL_{0}-10nlog\frac{R}{d_{0}}+\varepsilon \\ 10nlog\frac{R}{d_{0}}=P_{TX}-PL_{0}-RSS_{P_{entry}}+\varepsilon \\ log\frac{R}{d_{0}}=\frac{P_{TX}-PL_{0}-RSS_{P_{entry}}+\varepsilon }{10n}\\ R=d_{0}*10^{\left(\frac{P_{TX}-PL_{0}-RSS_{P_{entry}}+\varepsilon }{10n}\right)} \end{array}$$ (7)

where *d*_*0*_ is the distance between the AP and a reference point, *P*_*TX*_ is the AP transmit power, *PL*_*0*_ is the power loss at the reference point, *n* is the path loss exponent, and *ε* is a zero-mean Gaussian random variable caused by shadow fading. A low-pass filter can be used to suppress the shadowing part [18]. Similarly, the *r* value can be calculated by replacing ${RSS}_{P_{entry}}$ in (7) with ${RSS}_{P_{In\_RSSth}}$.

In this paper, we assume Doppler shift problem caused by high mobility can be mitigated by using the Doppler diversity. In [19], Doppler domain multiplexing communication structure is proposed to achieve the maximum Doppler diversity in time varying fading. Furthermore, the Doppler frequency offset estimation and compensation algorithms presented in [20, 21] can be used to alleviate the Doppler effects in high-speed environment.

The RSS value is monitored by MT periodically at time interval of *T*_*m*_ which is given by $\frac{D_{s}}{v}$second, where *v* is the measured MT’s velocity and *D*_*S*_ is a fixed sampling distance of 1 m [22]. *N* number of *RSS* samples is collected over the time interval of *ρ* and the median value is selected to determine the distance between MT and AP. This is to minimize the impact of RSS fluctuation. The median method [shown in (8)] is used instead of the mean method [23] because it overcomes a sudden large increase or decrease in the RSS value caused by an unintended factor. Therefore, *RSS* value is given as

$$RSS=Med\{{RSS}_{1},{RSS}_{2},\ldots {RSS}_{N}\}.$$ (8)

The number of samples *N* is adjusted dynamically to the MT’s traveling velocity expressed as

$$N=\left\lceil \frac{\rho }{T_{s}}\right\rceil$$ (9)

where *T*_*s*_ is RSS sampling time (*T*_*s*_ = 1 ms) and *ρ* = *K* * *T*_*m*_, *K* ∈ [0.1,0.9]. The maximum number of RSS samples is limited to 30 to avoid excessive sampling at low traveling speed. Once the *d*, *R*, and *r* values are obtained, angle *β* can be determined using the cosine law as follows:

$$\begin{array}{c} R^{2}=d^{2}+r^{2}–2drcos\;\beta \\ \beta =arccos\frac{d^{2}+r^{2}-R^{2}}{2dr} \end{array}$$ (10)

By knowing *β*, we can determine angle α by

$$\alpha =\pi -\beta \;,\;\frac{\pi }{2}<\beta <\pi .$$ (11)

Then, we can calculate the traveling distance *l*.

$$\begin{array}{c} r^{2}=r^{2}+l^{2}-2rlcos\alpha \\ l=2rcos\alpha \end{array}$$ (12)

From (11)

cosα=cos(π−β)=−cosβ (13)

Substituting (13) into (12) yields

$$\begin{array}{c} l=-2rcos\beta \\ \beta =arccos\left(-\frac{l}{2r}\right) \end{array}$$ (14)

Substituting (14) into (10) yields distance *l* as

$$\begin{array}{c} arccos\left(-\frac{l}{2R}\right)=arccos\frac{d^{2}+r^{2}-R^{2}}{2dr}\\ -\frac{l}{2r}=\frac{d^{2}+r^{2}-R^{2}}{2dr}\\ l=-\left(\frac{d^{2}+r^{2}-R^{2}}{d}\right)=\frac{R^{2}-r^{2}-d^{2}}{d} \end{array}$$ (15)

By applying the kinematic equation, *l* also can be expressed as

$$l=\frac{1}{2}cT_{WLAN}^{2}+v_{R}T_{WLAN}$$ (16)

where *c* denotes MT’s acceleration or deceleration rate. It can be determined by

$$c=\frac{v_{R}-v_{e}}{t_{d}}=\frac{v_{R}-v_{e}}{\left|t_{R}-t_{e}\right|}$$ (17)

where *t*_*R*_ and *t*_*e*_ represent the time of the MT passes through P_In_RSSth_ and P_entry_, respectively.

Next, we derive *d* from (15).

$$\begin{array}{c} ld=R^{2}-r^{2}-d^{2}\\ d^{2}+ld+\left(r^{2}-R^{2}\right)=0\\ d=\frac{-l+\sqrt{l^{2}-4\left(r^{2}-R^{2}\right)}}{2},\;d>0 \end{array}$$ (18)

Instead of using the time threshold similar to what were previously presented in [6, 15], we introduce a threshold for distance *d* known as *d*_*th*_ so that the MT can make handover decision by comparing the measured *d* value with *d*_*th*_ directly. We can derive *d*_*th*_ from (18) by replacing *l* with *l*_*th*_ and *d* with *d*_*th*_. It is given as

$$d_{th}=\frac{{-l}_{th}+\sqrt{l_{th}^{2}-4(r^{2}-R^{2})}}{2},\;d_{th}>0$$ (19)

where *l*_*th*_ is the traveling distance threshold. From (16), *l*_*th*_ for handover failure *l*_*thf*_ and unnecessary handover *l*_*thu*_ can be expressed as

$$l_{thf}=\frac{1}{2}cT_{i}^{2}+v_{R}T_{i}$$ (20)

$$l_{thu}=\frac{1}{2}c{\left(T_{i}+T_{o}\right)}^{2}+v_{R}(T_{i}+T_{o})$$ (21)

In constant speed (*c* = 0), *l*_*thf*_ = *vT*_*i*_ and *l*_*thu*_ = *v*(*T*_*i*_ + *T*_*o*_) which are identical to Yan et al.’s method as shown in (1) and (3) if both *P*_*f*_ and *P*_*u*_ are equal to 0. By substituting (20) into (19) yields *d*_*th*_ for handover failure *d*_*thf*_ as

$$d_{thf}=\frac{-(\frac{1}{2}cT_{i}^{2}+v_{R}T_{i})+\sqrt{{\left(\frac{1}{2}cT_{i}^{2}+v_{R}T_{i}\right)}^{2}-4(r^{2}-R^{2})}}{2}$$ (22)

Similarly, *d*_*th*_ for unnecessary handover *d*_*thu*_ can be determined by substituting (21) into (19) and it is given as

$$d_{thu}=\frac{-(\frac{1}{2}c{\left(T_{i}{+T}_{o}\right)}^{2}+v_{R}(T_{i}{+T}_{o}))+\sqrt{{\left(\frac{1}{2}c{\left(T_{i}{+T}_{o}\right)}^{2}+v_{R}(T_{i}{+T}_{o})\right)}^{2}-4(r^{2}-R^{2})}}{2}$$ (23)

**Fig 2:**
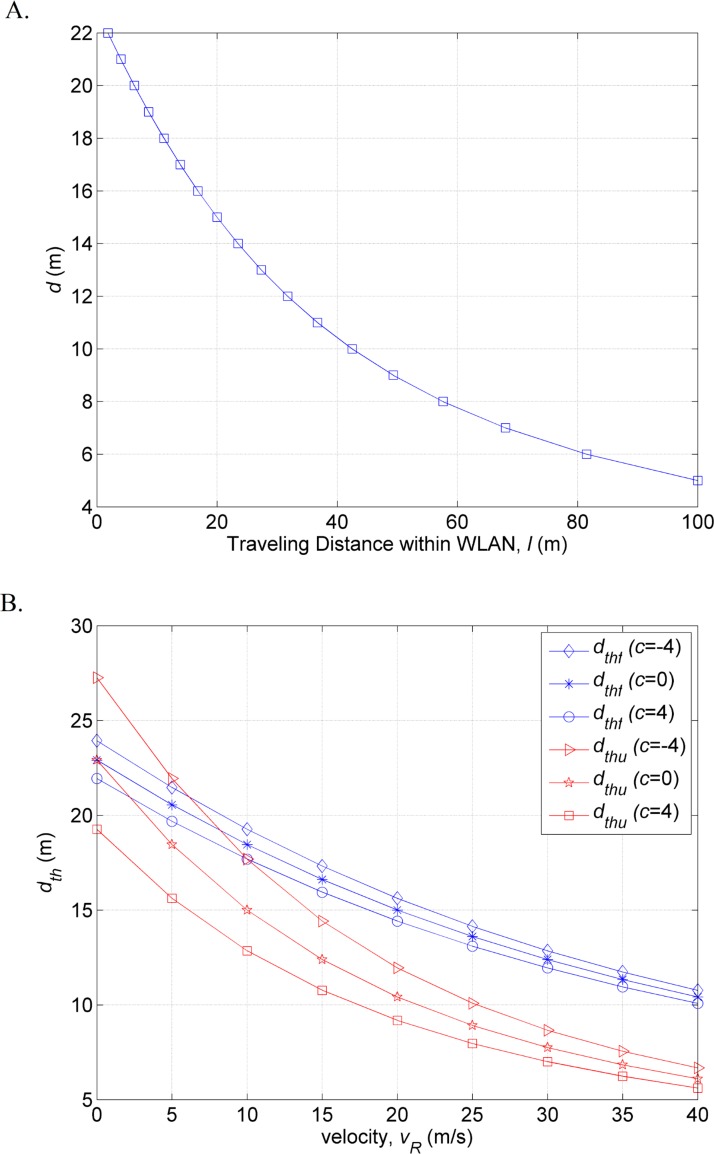
Relationship between (A) the *d* value and traveling distance *l* and (B) *d*_*th*_, acceleration and velocity.

The relationship between *d* and *l* is shown in Fig 2A, which shows that *d* inversely varies with *l*, indicating that the greater the traveling distance within WLAN coverage *l* is, the smaller is the *d* value. Referring to (22) and (23), both *d*_*thu*_ and *d*_*thf*_ are depending on the handover latency, acceleration or deceleration rate and velocity. By setting the handover latency (*T*_*i*_ and *T*_*o*_) at 1 second [6, 15], the correlation of *d*_*th*_ (*d*_*thu*_ and *d*_*thf*_), *c*, and *v*_*R*_ is shown in Fig 2B, where the *d*_*th*_ value decreases when the MT’s velocity increases or speeds up. The proposed method triggers a handover to the WLAN if and only if measured *d* value is less than estimated *d*_*th*_ value.

## Performance Evaluation

The scenario as shown in Fig 1 was simulated using OPNET. The WLAN cell is expected within the coverage of cellular network. We assume that the MT was connected to cellular network and seek for the availability of a WLAN. MT initiates the prediction process when the measured WLAN’s RSS value is greater than the predefined ${RSS}_{P_{entry}}$(MT reached point *P*_*entry*_). In the prediction process, MT calculates the *d*, *d*_*thf*_ and *d*_*thu*_ value based on the *v*_*R*_, *v*_*e*_, *t*_*R*_, *t*_*e*_, *R* and *r* values measured at point *P*_*entry*_ and *P*_*In_RSSth*_ (measured WLAN’s RSS value > *RSS*_*th*_). If calculated *d* value is less than the *d*_*th*_ value (*d*_*thf*_ and *d*_*thuI*_), MT triggers handover to WLAN cell. Otherwise, it remains connected to cellular network.

In this experiment, 10,000 random trajectories were generated for MT to cross the WLAN coverage at speeds from 11.11 to 41.66 m/s (40 to 150 km/h) in 2.22 m/s (8 km/h) increments. The simulation parameters listed in Table 1 were selected to observe the performance of the proposed method. The radius *r* is set as 50 m and the total handover latency for the handover into and out of the WLAN was 2 seconds as in [6, 15]. The traveling time within the WLAN coverage was nearly equal to the total handover latency when the MT velocity reached 150 km/h. As a result, unnecessary handover always occurred when the speed of the MT was higher than 150 km/h.

**Table 1:** Simulation parameters.

**Parameter**	**Value**
AP physical characteristics	HT PHY 2.4GHz (802.11n)
RSS at P_entry_, *RSS*_*Pentry*_	-80.2 dBm
RSS threshold, *RSS*_*th*_	-79.3 dBm
Number of Trajectory	10,000
Adaptive sensing time interval	1/*v*
*R*	55 m
*r*	50 m [6]
*v*	11.11 to 41.66 m/s
*c* (first scenario)	0
*c* (second scenario)	Random (1 ~ 5 m/s^2^)
*t*_*s*_*—t*_*PIn_RSSth*_	0.1, 0.2 s
*K*	0.2
*T*_*s*_	0.001 s
*T*_*i*_	1 s [6, 15]
*T*_*o*_	1 s [6, 15]
Time threshold for handover failure	*T*_*i*_ [6, 15]
Time threshold for unnecessary handover	*T*_*i*_ + *T*_*o*_ [6, 15]
Tolerable handover failure probability, *P*_*f*_	0
Tolerable unnecessary handover probability, *P*_*u*_	0

The experiment was simulated within two scenarios. First scenario is that the MT crosses the WLAN cell in random direction with speed remaining fixed (*c* = 0) within the WLAN coverage [6, 15]. The second scenario is that the MT crosses the WLAN coverage in random direction with smooth acceleration in the range of 1 m/s^2^ to 5 m/s^2^. The deceleration condition is not critical to handover failure and unnecessary handover because it offers MT longer traveling time within the WLAN coverage. Therefore, deceleration condition was not considered in this experiment.

**Fig 3:**
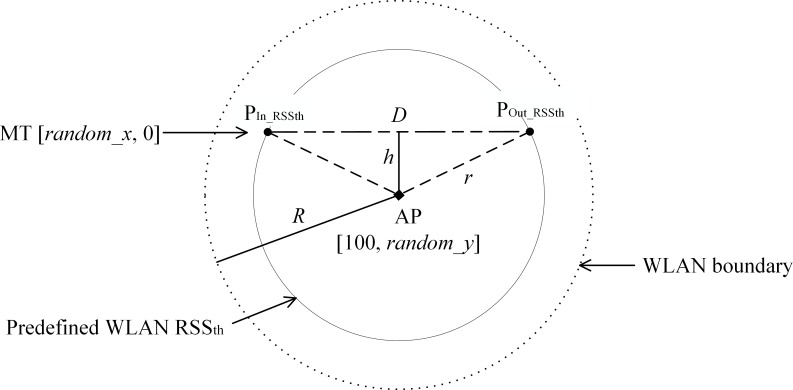
Scenario of MT traveling trajectory in the WLAN coverage.

In order to generate random trajectories within the WLAN coverage, a random AP coordinate was generated in this experiment. The *x* coordinate of the AP was fixed at 100, but the *y* coordinate (*random_y*) was a random value within the range of -*r* to *r* (*r* = 50). This is to ensure that all the trajectories are crossing the WLAN coverage. The distance of each trajectory is 200 m. The actual traveling distance within the WLAN coverage (*D*) is expressed as [6]

$$D=2\sqrt{r^{2}-h^{2}}$$ (24)

where *h = |AP y coordinate*–*MT y coordinate|*, as shown in Fig 3. The MT *x* coordinate (*random_x*) was randomly set from 0 to 30 m (*random_x* ∈ [0,30]) to create a different starting point for each MT when it moves toward the WLAN coverage.

The handover failure and unnecessary handover occur if the actual traveling time within the WLAN coverage (*T*_*WLAN*_) is less than *T*_*i*_ and *T*_*i*_+*T*_*o*_, respectively. The actual traveling time within the WLAN coverage for first scenario (*c* = 0) can be determined by $\frac{D}{v}$. In the second scenario (*c* = 1~5 m/s^2^), the actual traveling time within the WLAN coverage can be calculated by

$$\begin{array}{c} D=\frac{1}{2}cT_{WLAN}^{2}+v_{R}T_{WLAN}\\ \frac{1}{2}cT_{WLAN}^{2}+v_{R}T_{WLAN}-D=0\\ T_{WLAN}=\frac{-v_{R}+\sqrt{v_{R}{}^{2}-2cD}}{c},\;c>0 \end{array}$$ (25)

**Fig 4:**
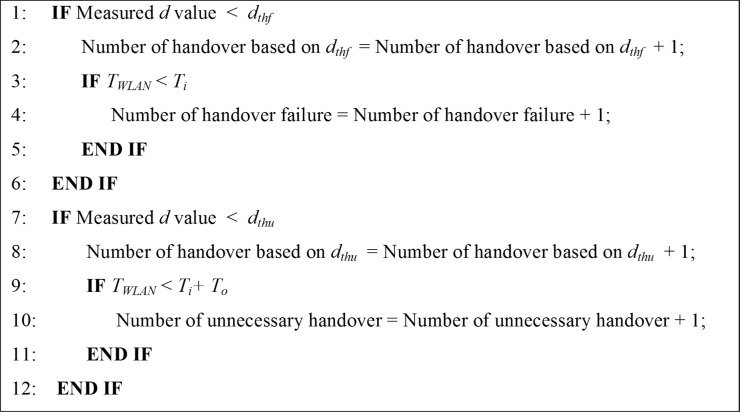
Pseudo code for determine number of handover failures and unnecessary handovers.

The pseudo code of determine the number of handover failures and unnecessary handovers is shown in Fig 4.

**Fig 5:**
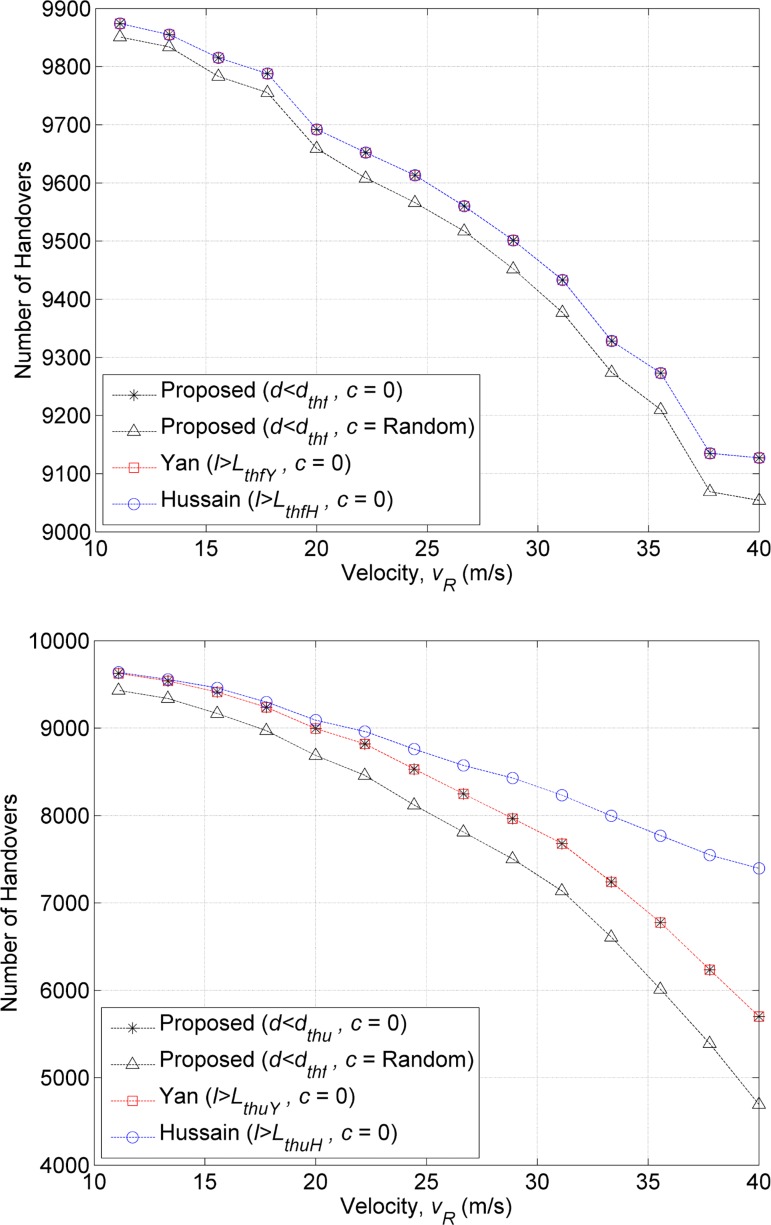
Total number of handovers to the WLAN based on (A) handover failure threshold and (B) unnecessary handover threshold.

The performance of the proposed method is compared against the existing methods from Yan et al. [6] and Hussain et al. [15]. Fig 5 shows the total number of handovers performed by the existing methods and the proposed methods for the speeds from 11.11 to 41.66 m/s. Fig 5A shows that in the first scenario, all the methods have identical number of handovers based on the handover failure thresholds (*d*_*thf*_, *L*_*thfY*_, and *L*_*thfH*_, respectively) defined by each method. In terms of the triggered handovers based on the unnecessary handover threshold (*d*_*thu*_, *L*_*thuY*_, and *L*_*thuH*_, respectively), the method of Hussain et al. has higher number of handovers than the other methods (Fig 5B). In the second scenario, the number of handovers attained by the proposed method is lower than the first scenario. This is due to the smaller *d*_*th*_ value as the MT accelerates (shown in Fig 2B). However, the previous methods [6, 15] have same number of handovers as first scenario because these methods do not consider speed change and assumed all the MTs cross the WLAN coverage in constant speed.

**Fig 6:**
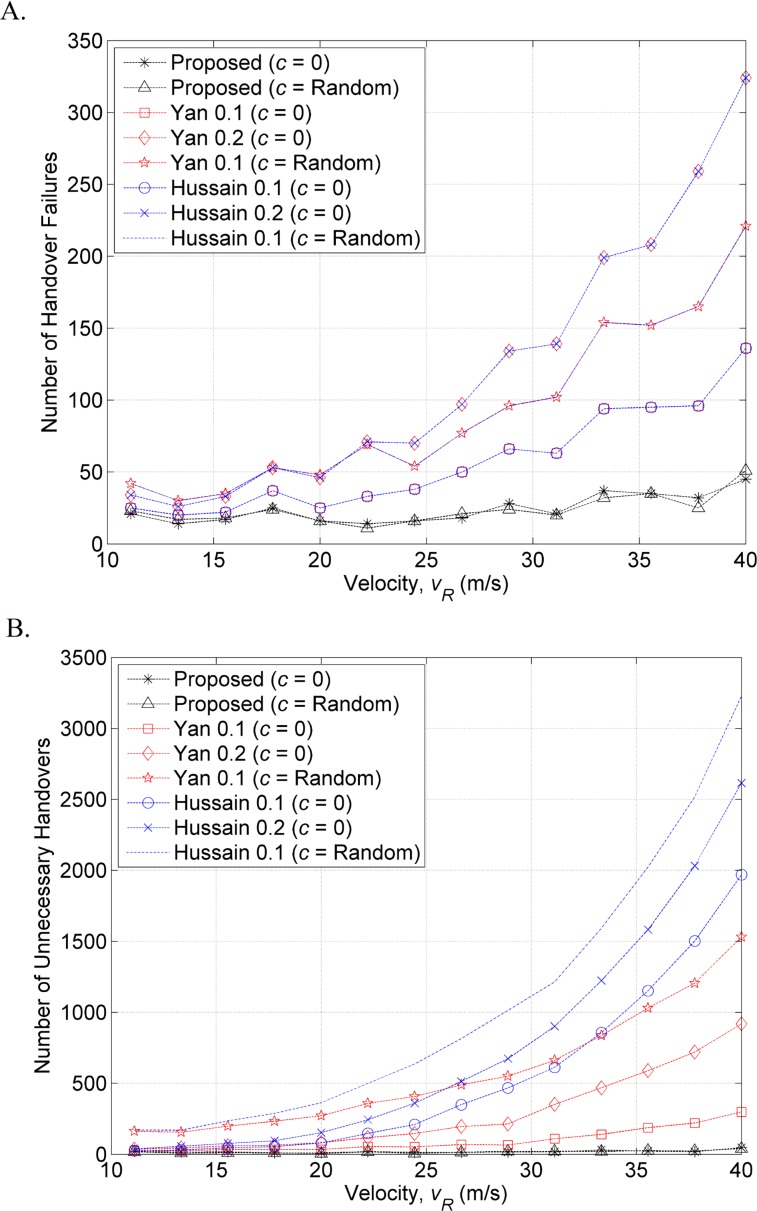
Number of (A) handover failures and (B) unnecessary handovers.

The number of handover failures and unnecessary handovers were determined by applying different values of $t_{s}-t_{P_{In\_RSSth}}$ (in Table 1) to the previous methods [6, 15]. The simulation results shown in Fig 6 show that the larger the value of $t_{s}-t_{P_{In\_RSSth}}$ is, the higher is the number of handover failures and unnecessary handovers when the MT traveling speed increases. However, the proposed method is not affected by the $t_{s}-t_{P_{In\_RSSth}}$value because it completes the traveling distance prediction process when MT reaches P_In_RSSth_. In contrast, the previous works initiated the prediction process after the MT reached P_In_RSSth_.

In the second scenario (*c* = Random), the traveling time in WLAN coverage is always less than the time in the first scenario due to the acceleration. Therefore, the previous methods have even more handover failures and unnecessary handovers in the second scenario owing to the previous methods assuming MT remains at constant speed within WLAN coverage. Fig 6 shows the number of handover failures and unnecessary handovers obtained by the proposed method are much lower than the previous methods.

**Fig 7:**
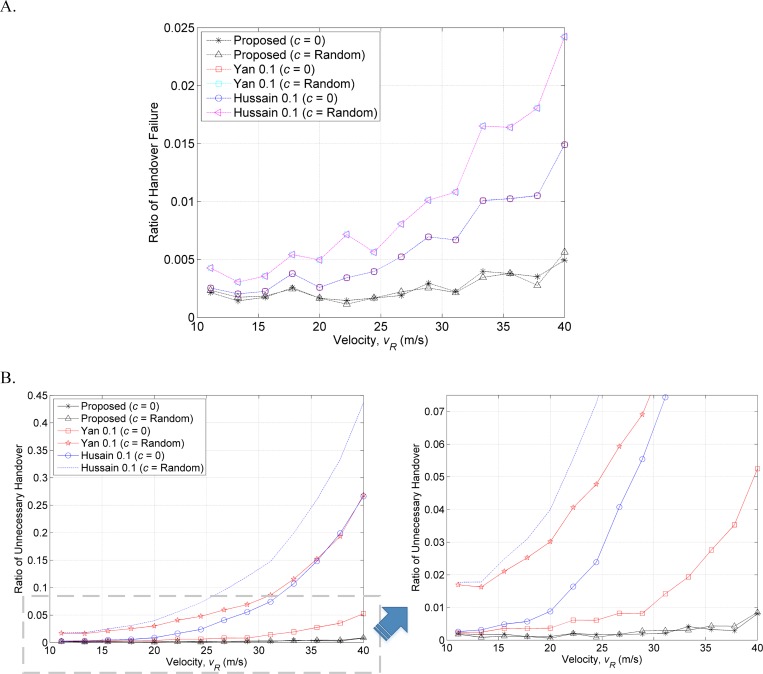
Ratio of (A) handover failures and (B) unnecessary handovers to the total number of handovers.

For better comparison of the performance results, we divided the number of handover failures and unnecessary handovers by the total number of handovers. Fig 7 shows that the ratio of handover failures and unnecessary handovers of the proposed methods are below 0.005 and 0.01 for both scenarios. It outperforms the previous methods that have been presented in [**6**, **15**]. The handover failures and unnecessary handovers ratio of the proposed method rise while the MTs travel at higher speed. This is due to the fewer RSS samples collected by MT at higher speed affects the accuracy of RSS measurements and distance prediction. The summary of the performance comparison results is listed in Table **2**. The results show that the proposed method has successfully reduced the probability of handover failures and unnecessary handovers to the WLAN by up to 66.7% and 96.3% in first scenario (*c* = 0), and 80.0% and 97.7% in second the scenario (*c* = 1~5 m/s^2^) compared with the previous methods.

**Table 2:** Summary of performance comparison.

**Methods**	**Yan et al.[6]**		**Hussain et al.[15]**		**Proposed**	
$t_{s}-t_{P_{In\_RSSth}}$ (s)	0.1	0.1	0.1	0.1	Not Applicable	Not Applicable
*c* (m/s^2^)	0	Random	0	Random	0	Random
Ratio of the number of handover failures to the total number of handovers	< 0.015	< 0.025	< 0.015	< 0.025	< 0.005	< 0.005
Ratio of the number of unnecessary handovers to the total number of handovers	< 0.055	< 0.270	< 0.270	<0.430	< 0.010	< 0.010

## Conclusion

This paper has presented a new vertical handover method to optimize the utilization of WLAN in high-speed environment. The proposed method overcomes the imperfections and limitations of previous methods. The simulation results show that the proposed method performs better than the previous methods. It keeps the probability of handover failure and unnecessary handover below 0.5% and 1% respectively. The proposed method can be applied for all handovers from macro-cell, such as Long Term Evolution (LTE), Worldwide Interoperability for Microwave Access (WiMAX) or Universal Mobile Telecommunications System (UMTS) to WLAN. With the proposed method, the user can benefit more from WLAN cells with high bandwidth and low access cost, which can improve the quality of service and the cost effectiveness.

In future, we will focus on fast and robust authentication to further improve the performance of handover from macro-cell to WLAN in high mobility scenario. An efficient authentication scheme can minimize the handover latency, packet loss and communication overhead [24]. The recent authentication schemes and protocol designs can be found in [25–29].

## Supporting Information

S1 Dataset Data of simulation results. A: Relationship between the *d*, acceleration and velocity (Fig 2A). B: Relationship between *d*_*th*_, acceleration and velocity (Fig 2B). C: Total number of handover to the WLAN based on the handover failure threshold (Fig 5A). D: Total number of handover to the WLAN based on the unnecessary handover threshold (Fig 5B). E: Number of handover failures (Fig 6A). F: Number of unnecessary handovers (Fig 6B). G: Ratio of handover failures to the total number of handovers (Fig 7A). H: Ratio of unnecessary handovers to the total number of handovers (Fig 7B).
(XLSX)
